# Medical education under siege: the war’s impact on medical and paramedical Sudanese students

**DOI:** 10.1186/s12909-025-07457-4

**Published:** 2025-07-01

**Authors:** Toga Khalid Mohamed, Tibyan O. Abdalla, Salma G. Ahmed, Alanood Elnaeem, Tanzeel M. A. Omer, Taiba H. Hassan, Naeema S. Mamoun, Maha A. H. Musa, Ahmed E. B. Zaidan, Safaa M. A. Omer, Abdalla O. ElKhawad, Kannan O. Ahmed

**Affiliations:** 1 Department of Clinical Pharmacy, Faculty of Pharmacy, University of Medical Sciences & Technology, Khartoum, Sudan; 2 Department of Pharmacology, Faculty of Pharmacy, University of Medical Sciences and Technology, Khartoum, Sudan; 3 Department of Clinical Pharmacy, Faculty of Pharmacy, National University, Khartoum, Sudan; 4 Faculty of Pharmacy, University of Medical Sciences & Technology, Khartoum, Sudan; 5 Faculty of Pharmacy, The National Ribat University, Khartoum, Sudan; 6 Department of Clinical Pharmacy, Faculty of Pharmacy, Ahfad University for Women, Khartoum, Sudan; 7 Faculty of Dentistry, Nile University, Khartoum, Sudan; 8 Department of Pharmacy Practice, College of Pharmacy, National University of Science and Technology, Muscat, Oman; 9 Department of Clinical Pharmacy and Pharmacy Practice, Faculty of Pharmacy, University of Gezira, Wad Medani, Sudan

**Keywords:** Medical education, Medical students, Paramedical, Sudan, War

## Abstract

**Background:**

Medical education is pivotal in shaping healthcare systems globally. In Sudan, the war that erupted on the 15th of April 2023 has imposed significant implications and challenges for medical education in Sudan. The continuity and quality of medical education remains fragile, especially in the face of ongoing armed conflict. This study aims to examine the impact of the Sudanese war on medical students’ educational experiences.

**Materials and methods:**

This cross-sectional quantitative survey assessed the impact of the Sudan war on medical students’ education. From April to November 2023, data were collected using a structured, self-administered online questionnaire covering demographics, psychological impact, educational access, satisfaction, wartime considerations, and barriers to education. A total of 245 undergraduate students from medical faculties in Khartoum and Gezira states were recruited through convenience sampling. Data analysis was performed using SPSS version 23. Descriptive statistics and non-parametric tests (Mann–Whitney U and Kruskal–Wallis H) were used for group comparisons, and weighted means were calculated for key indicators. A p-value < 0.05 was considered statistically significant.

**Results:**

The study included 245 medical students, predominantly female (81.6%) and aged 21–23 (59.6%), with the majority enrolled in private institutions. Pharmacy students represented 53.9% of respondents, and most were displaced (93.1%), primarily to Egypt, Saudi Arabia, and the UAE. Psychological distress was moderate to high among participants (weighted mean = 3.71 ± 1.11), with symptoms including anxiety, depression, or PTSD. Educational access was severely disrupted (weighted mean = 3.86 ± 1.31), with nearly half reporting very high disruption. While some universities implemented online learning or relocation strategies, many students reported dissatisfaction with these efforts, especially regarding clinical training and faculty interaction (overall satisfaction mean = 1.69 ± 1.66). Students considered various coping strategies, such as seeking scholarships abroad or part-time work. Major barriers to continuing education included psychological impact (49.8%), financial hardship (46.9%), and institutional shortcomings (43.7%). Displaced students outside Sudan faced significantly greater challenges in accessing education (*p* = 0.047) and reported lower satisfaction with clinical training (*p* = 0.016), while professor interaction was significantly better among students inside Sudan (*p* = 0.030).

**Conclusion:**

This study elucidates the significant ramifications of armed conflict on the psychological health and educational experiences of medical students in Sudan. Elevated levels of psychological distress and considerable interruptions to both education and clinical training emphasize the pressing necessity for targeted interventions. A collaborative approach that integrates educational institutions, policymakers, and mental health practitioners is essential to enhance student support and guarantee the continuity of education. These efforts are vital to safeguarding the future quality of healthcare in conflict-affected regions.

**Clinical trial number:**

Not applicable.

## Introduction

Medical education plays a crucial role in shaping and enhancing healthcare systems worldwide. In a low-income country like Sudan, despite noticeable improvements in medical education in recent years, enduring challenges remain. Sudan boasts approximately 66 medical institutions, both public and private, positioning it as one of Africa’s leading countries in medical education [1]. However, the quality of healthcare in Sudan remains fragile. Before the conflict erupted in April 2023, Sudan struggled with inadequate hospital conditions, training facilities, and a shortage of medical professionals. The conflict has exacerbated these issues, resulting in the collapse of the country’s healthcare system, with two-thirds of medical facilities rendered inoperative since the outbreak of war [2]. Instances of violence against healthcare workers, including killings and abductions, have been documented [3]. Similarly, the educational sector has been severely impacted by threats, infrastructure destruction, and shortages of resources. A local study investigated the damage inflicted on medical schools in conflict zones, uncovering that all institutions suffered, particularly private faculties, which bore 70% of the damage. Furthermore, some institutions were repurposed as military centers, while others were robbed [4].

Many studies worldwide have investigated the effects of wars and political instability on medical education. For example, the Russia-Ukraine War significantly altered the standards of medical education within the country. Bombings destroyed crucial infrastructure in Ukraine’s medical facilities, resulting in the loss of educators, medical staff, and students [5]. This has had enduring consequences on academic endeavors, including comprehensive instruction and syllabus coverage. Presently, domestic and international students from Ukraine are internally displaced and seeking education in Europe, particularly in Germany, Italy, and Spain [6]. Similarly, Iraq has witnessed a decline in educational standards in medical schools since the American invasion in 2003. Among 197 surveyed medical students, 62% expressed concerns about safety due to violent insecurity, with 56% intending to leave the country after graduation [7]. This migration adds to the scarcity of university faculty due to the documented emigration of professors. Additionally, the war in Liberia from 1980 to 2003 had a detrimental effect on medical education due to facility destruction and medical supplies theft [8]. In Syria, medical students allege that the quality of education, training, and research is significantly impacted by the ongoing war [9].

Amidst the challenges, many medical schools in Sudan have transitioned to online education, placing pressure on teachers and students to continue their work amidst attacks. Furthermore, internet service instabilities have hindered students in rural areas from attending classes [10]. The significance of war’s influence on medical education in Sudan is profound, impacting numerous generations of medical students. Despite its importance, only a limited number of studies address the issue. This study seeks to bridge this knowledge gap by examining how the ongoing war in Sudan has affected the learning experiences of medical students in conflict-affected regions, specifically in terms of psychological well-being, educational access, and institutional response.

## Materials and methods

### Study design, setting, and duration

This study employs a cross-sectional quantitative survey to investigate the impact of war in Sudan on the educational experiences of medical students attending universities in conflict-affected regions. The war erupted in Khartoum on the 15th of April 2023, and extended to involve Gezira State, located 116 km from the capital, on the 17th of December 2023. The list of universities eligible for inclusion in the study was identified through the World Directory of Medical Schools database [11]. The data collection period spanned from April 2023 to November 2023.

### Study population and sampling

The study recruited undergraduate students from five faculties (Medicine, Pharmacy, Dentistry, Nursing, and Medical Laboratory Sciences) in Khartoum and Gezira states. Participants from non-medical faculties and other universities outside Khartoum and Gezira state were excluded from the study.

Due to the absence of institutional student email addresses, along with challenges in direct recruitment and access limitations caused by war, convenience sampling was used. An initial sample size of 384 medical students was obtained considering an unknown population, a confidence level of 95%, a 5% margin of error, and a proportion of 50%. A final sample size of 245 medical students was achieved accounting for a response rate of 63.8%. The response rate was deemed low due to disruptions caused by the war, including limited internet connectivity, displacement of students, and the inaccessibility of educational institutions, which significantly impacted the recruitment process.

### Data collection methods and tools

A structured, self-administered questionnaire was used for data collection. The questionnaire domains were developed in accordance with previous literature [7, 12]. The questionnaire was also reviewed by two university professors to ensure content suitability. A pilot study was conducted with 32 medical students selected through convenience sampling to assess the clarity, wording, and comprehension of the questionnaire items. Minor wording adjustments were made based on their feedback, without altering the structure or content of the survey. These participants were included in the final sample. A Crohnbach alpha value of 0.7 was indicative of the questionnaire’s internal consistency and reliability.

The questionnaire was distributed as an online-based Google Form in English language to the participants on various social media platforms (Telegram, Facebook, X, WhatsApp). To ensure data completeness, the online survey (Google Form) was designed such that participants could not submit their responses unless all questions were answered. As a result, there were no missing or incomplete responses in the dataset. Responses were collected using a 5-point Likert scale in multiple sections.

Also, the Likert scale responses ranged from 1 to 5, where each number represented varying degrees of impact or satisfaction. In some questions, the response options also included a “Not applicable” category for cases where no solution was employed or the situation did not apply. In regard to the Likert scale interpretation, **1–2**: Indicated a low to very low impact or dissatisfaction, **3**: Indicated a neutral response or moderate impact, and **4–5**: Indicated a high to very high impact or satisfaction. It should be noted that in some cases, the responses were categorized into **0**: “Not applicable” or “No solution employed” to ensure clarity and prevent bias in interpretation. For scoring, the Likert scale responses were numerically converted, with each response assigned a value ranging from 1 to 5, where 1 represented “very low impact” or “very dissatisfied” and 5 represented “very high impact” or “very satisfied.” To calculate the weighted mean, the values for each response option were multiplied by the corresponding frequency of responses, and the results were then summed and divided by the total number of respondents. For interpretation, a mean score greater than 3 was considered to represent a high impact or satisfaction, while a mean score below 3 indicated a low impact or dissatisfaction.”

The questionnaire was composed of several domains:

1. Demographics (age, gender, faculty, academic year, university, years in medical school, displacement status and location).
2. Psychological impact (war-related symptoms).
3. Educational access (accessibility to medical education during the current war period and the solutions employed by the Sudanese universities).
4. Student satisfaction (satisfaction level with the solutions implemented by their universities).
5. Considerations during war (students’ considerations during the war period including dropout likelihood, part-time jobs, career shifts, and external scholarships).
6. Barriers to medical education.

### Data management and analysis

The obtained data was organized and refined using Microsoft Excel. The data analysis was performed using the Statistical Package for Social Sciences- SPSS version 23. Descriptive statistics was used for the presentation of data in the form of frequencies and percentages. An overall weighted mean was used to determine the extent of psychological impact, disruption to educational access, medical students’ satisfaction, and overall students’ considerations. A comparative analysis was performed using non-parametric methods due to the non-normal distribution and ordinal nature of the variables. The Mann–Whitney U test was applied to compare the distributions between students inside and outside Sudan across six variables: psychological impact, access to education, university solutions, clinical training, professor interaction, and internet quality. A p-value of < 0.05 was considered statistically significant. The Mann–Whitney U test was used for binary group comparisons, while the Kruskal–Wallis H test was applied for comparisons involving three or more groups. Additionally, pie charts, tables, bar plots, and bar charts were used for data visualization.

## Results

**Fig. 1:**
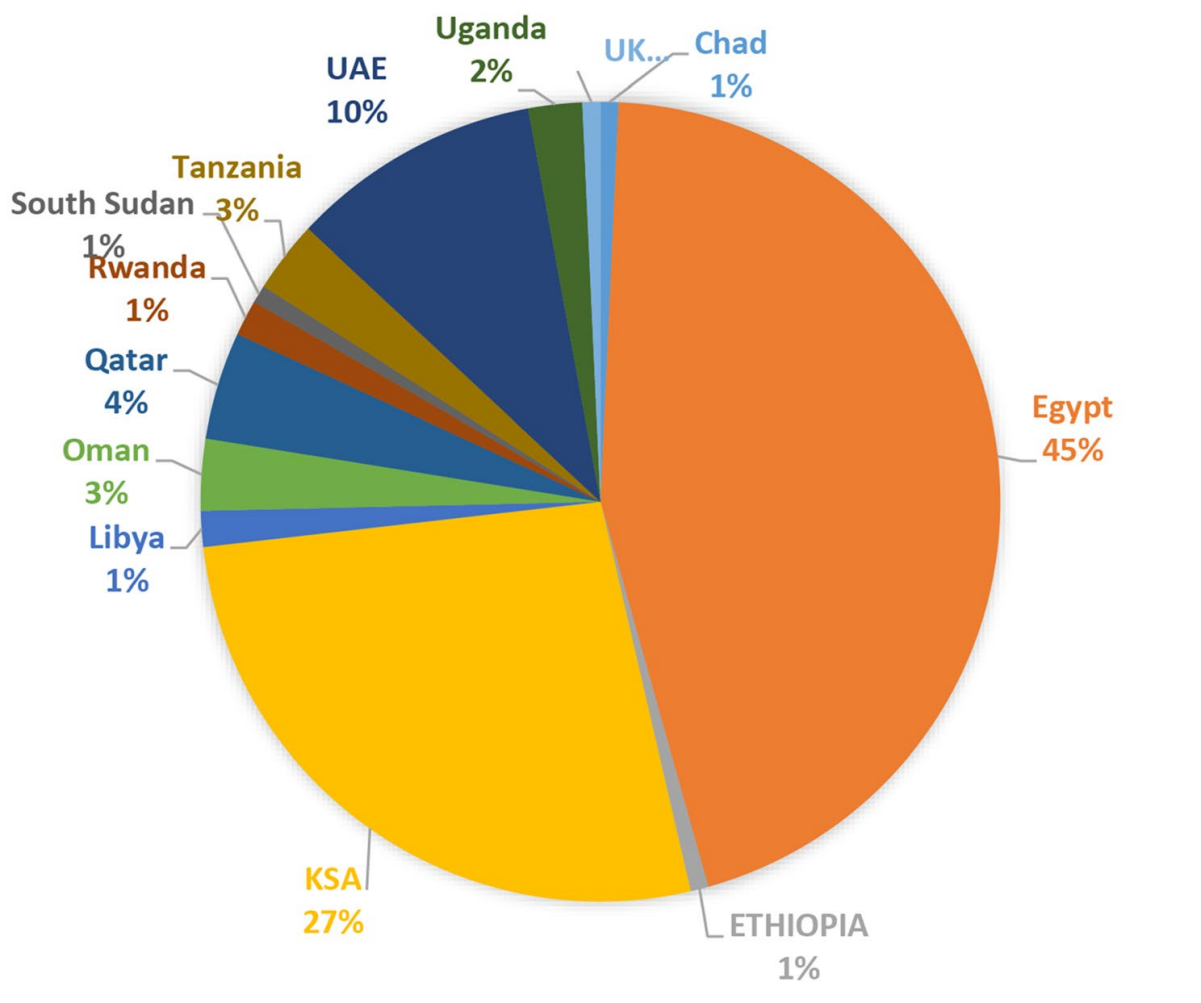
Participants’ distribution according to displacement location

Most of the study participants were in their early to mid-20s, with 59.6% aged 21–23. Females made up the majority (81.6%) of the participants. In terms of academic faculty, pharmacy was the most represented (53.9%). Furthermore, 39.2% of participants were in their fifth year of study, whereas only 2.9% were in their first year. The demographics are outlined in Table 1. The majority of the participants were enrolled in private institutions. The University of Medical Sciences and Technology (UMST) had the highest representation (29.8%), followed by Ahfad University for Women (25.3%), with the University of Khartoum having a smaller representation compared to both (5.3%) as shown in Fig. 1.

**Table 1:** Participants demographics (*n* = 245)

	*N* (%)
**Age**	
18–20	41 (16.7)
21–23	146 (59.6)
24–26	51 (20.8)
27 and more	7 (2.9)
**Gender**	
Male	45 (18.4)
Female	200 (81.6)
**Faculty**	
Medicine	83 (33.9)
Dentistry	22 (9.0)
Pharmacy	132 (53.9)
Medical Laboratory Sciences	6 (2.4)
Nursing	2 (0.8)
**Academic Year**	
Year 1	7 (2.9)
Year 2	26 (10.6)
Year 3	53 (21.6)
Year 4	54 (22.0)
Year 5	96 (39.2)
Year 6	9 (3.7)
**Mean number of years in medical faculties given impact of war**,** political instability and COVID-19**	
Starting year	2018.75
Graduation year	2025.19
Mean number of years of medical education	6.44
**Displacement**	
Yes	228 (93.1)
No	17 (6.9)
**Displacement Location**	
Inside Sudan ( other states)	78 (31.8)
Outside Sudan	148 (60.4)
Not Displaced	19 (7.8)

**Fig. 2:**
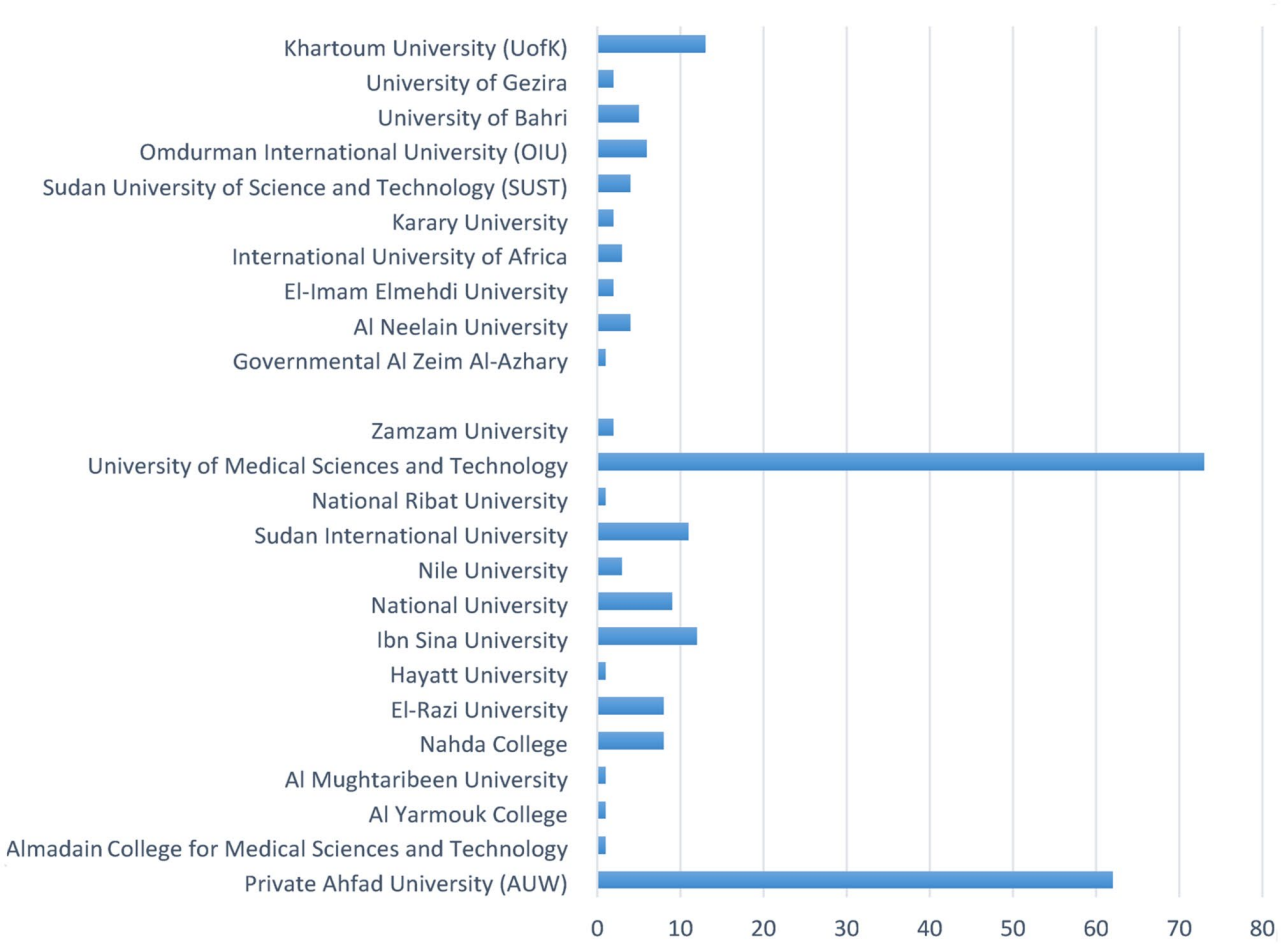
Distribution of university categories in the study

Regarding the year of enrollment, the largest proportion of students were enrolled in the year 2018 (31.4%), followed by 2019 (20.0%), while earlier and later years, such as 2014, 2015, 2020, and 2023, had minimal representation. Moreover, the majority were expected to graduate in 2024 (20.8%), followed by 2026 (17.1%) and 2025 (14.7%), though 14.7% had no defined graduation year. On average, students take 6.44 years to graduate (see Table 1). Additionally, 93.1% of participants were displaced, with (60.4%) being displaced outside Sudan. The highest percentage of study participants were displaced to Egypt (45%), followed by KSA (27%) and the UAE (10%) as shown in Fig. 2.

The findings show that the psychological impact of war on participants was moderate to high, with a weighted mean of 3.71 ± 1.11. Primarily, 27.8% of participants experienced moderate psychological distress due to war, 29.0% reported high psychological distress, and 29.8% faced very high psychological impact. Among respondents, 6.9% reported no psychological impact, while 93.1% reported at least one form of psychological distress, including PTSD (15.5%), stress and anxiety (43.7%), depression (29.4%), distraction and lack of focus (3.3%) and two or more of reported symptoms (1.2%). This is shown in Table 2.

**Table 2:** Psychological impact of war on participants (*n* = 245)

		*N* (%)
To what extent did the current war impact your mental health and psychological status?	1 (very low impact)	10 (4.1)
	2 (low impact)	23 (9.4)
	3 (moderate impact)	68 (27.8)
	4 (high impact)	71 (29.0)
	5 (very high impact)	73 (29.8)
**Overall Weighted Mean = 3.71 ± 1.11**		
Note the main Psychological impact inflicted on you during the war period?	Stress and anxiety	107 (43.7)
	Depression	72 (29.4)
	Post-Traumatic stress disorder (PTSD)	38 (15.5)
	Distraction and Lack of Focus	8 (3.3)
	Two or more of the above symptoms	3 (1.2)
	No impact	17 (6.9)

In regards to accessibility to medical education during the current war period, 46.1% of participants reported a very high level of disruption in access to medical education and training. Additionally, 17.6% reported moderate disruption. The overall weighted mean was 3.86 ± 1.31, reflecting a substantial level of disruption caused by the war. In response, 46.9% of universities adopted online classes to ensure education continuity, while 16.3% relocated to other countries and 5.7% moved to other Sudanese states. However, 27.8% of participants reported that no solutions were implemented, and 3.3% mentioned alternative measures, as outlined in Table 3. Overall, medical students expressed dissatisfaction with the solutions implemented by their universities to maintain educational continuity, with 14.3% reporting that they were very dissatisfied. In terms of clinical and hospital training, only 5.7% of students expressed satisfaction, and 5.3% were very satisfied. In contrast, 15.1% reported being very dissatisfied with the quality of clinical and hospital training.

**Table 3:** Impact of war on access to education and training (*n* = 245)

		*N* (%)
To what extent did the current war affect your access to medical education and training?	1 (Very low impact)	20 (8.2)
	2 (Low impact)	21 (8.6)
	3 (Moderate impact)	43 (17.6)
	4 (High impact)	48 (19.6)
	5 (Very high impact)	113 (46.1)
**Overall Weighted Mean = 3.86 ± 1.31**		
What solutions were employed by your university for education continuity during war period?	Move university to another Sudanese state	14 (5.7)
	Move university to another country	40 (16.3)
	Start online classes	115 (46.9)
	No solutions were employed	68 (27.8)
	Other solutions	8 (3.3)

A similar level of dissatisfaction was observed regarding the ability to interact with professors, with 18% of participants reporting being very dissatisfied. Additionally, internet connectivity and platforms used for delivering lectures posed a challenge, with 21.2% of participants being greatly dissatisfied. The overall weighted mean was 1.69 ± 1.66, as shown in Table 4.

**Table 4:** Satisfaction of medical students to solutions implemented by their universities during war period. (*n* = 245)

		*N* (%)
How satisfied are you with the solutions employed by your university to help continue education and training during war period?	0 (Not applicable, no solution employed)	76 (31)
	1 (Very Dissatisfied)	35 (14.3)
	2 (Dissatisfied)	31 (12.7)
	3 (Neutral)	53 (21.6)
	4 (Satisfied)	28 (11.4)
	5 (Very Satisfied)	22 (9.0)
How satisfied are you with the quality of clinical and hospital training (if any) during war period?	0 (Not applicable, no solution employed)	124 (50.6)
	1 (Very Dissatisfied)	37 (15.1)
	2 (Dissatisfied)	26 (10.6)
	3 (Neutral)	31 (12.7)
	4 (Satisfied )	14 (5.7)
	5 (Very Satisfied)	13 (5.3)
How satisfied are you with the ability to interact with professors and educators during war period?	0 (Not applicable, no solution employed)	73 (29.8)
	1 (Very Dissatisfied)	44 (18)
	2 (Dissatisfied)	35 (14.3)
	3 (Neutral )	50 (20.4)
	4 (Satisfied)	22 (9.0)
	5 (Very Satisfied)	21 (8.6)
How satisfied are you with the quality of the internet connectivity and platforms used for delivering lectures during war period?	0 (Not applicable, no solution employed)	79 (32.2)
	1 (Very Dissatisfied)	52 (21.2)
	2 (Dissatisfied)	32 (13.1)
	3 (Neutral)	41 (16.7)
	4 (Satisfied)	20 (8.2)
	5 (Very Satisfied)	21 (8.6)
**Overall Weighted Mean = 1.69 ± 1.66**		

In an attempt to determine medical students’ considerations that were posed as a result of war, the responses were variable. When asked about the possibility of dropout from medical school, 17.1% of participants reported thoroughly considering it while 24.1% did not consider dropout as an option given the current circumstances. The highest percentage (33.5%) of participants reported considering taking on a part-time job, while (13.1%) were not considering it as an option. 31.8% considered seeking a scholarship or admission to a university outside Sudan to continue their medical education. Nonetheless, 20.4% did not consider applying for a scholarship. 29.8% of participants did not attempt to consider a non-medical or alternative degree as an option for continuation of studies. This is indicated in Table 5. Overall, the weighted mean was 3.13 ± 1.47.

**Table 5:** Medical students considerations during the war period (*n* = 245)

		*N* (%)
To what extent did you consider dropout from medical school and university?	1 (Very small extent)	59 (24.1)
	2 (Small extent)	30 (12.2)
	3 (Neutral)	67 (27.3)
	4 (Large extent)	47 (19.2)
	5 (Very large extent)	42 (17.1)
To what extent did you consider starting a part-time job?	1 ( Very small extent)	32 (13.1)
	2 (Small extent)	22 (9.0)
	3 (Neutral)	57 (23.3)
	4 (Large extent)	52 (21.2)
	5 (Very large extent)	82 (33.5)
To what extent did you consider a scholarship or admission to a university outside Sudan?	1 (Very small extent)	50 (20.4)
	2 (Small extent)	29 (11.8)
	3 (Neutral)	50 (20.4)
	4 (Large extent)	38 (15.5)
	5 (Very large extent)	78 (31.8)
To what extent did you consider a nonmedical or alternative degree as a career choice?	1 (Very small extent)	73 (29.8)
	2 (Small extent)	33 (13.5)
	3 (Neutral)	59 (24.1)
	4 (Large extent)	37 (15.1)
	5 (Very large extent)	43 (17.6)
**Overall Weighted Mean = 3.13 ± 1.47**		

**Fig. 3:**
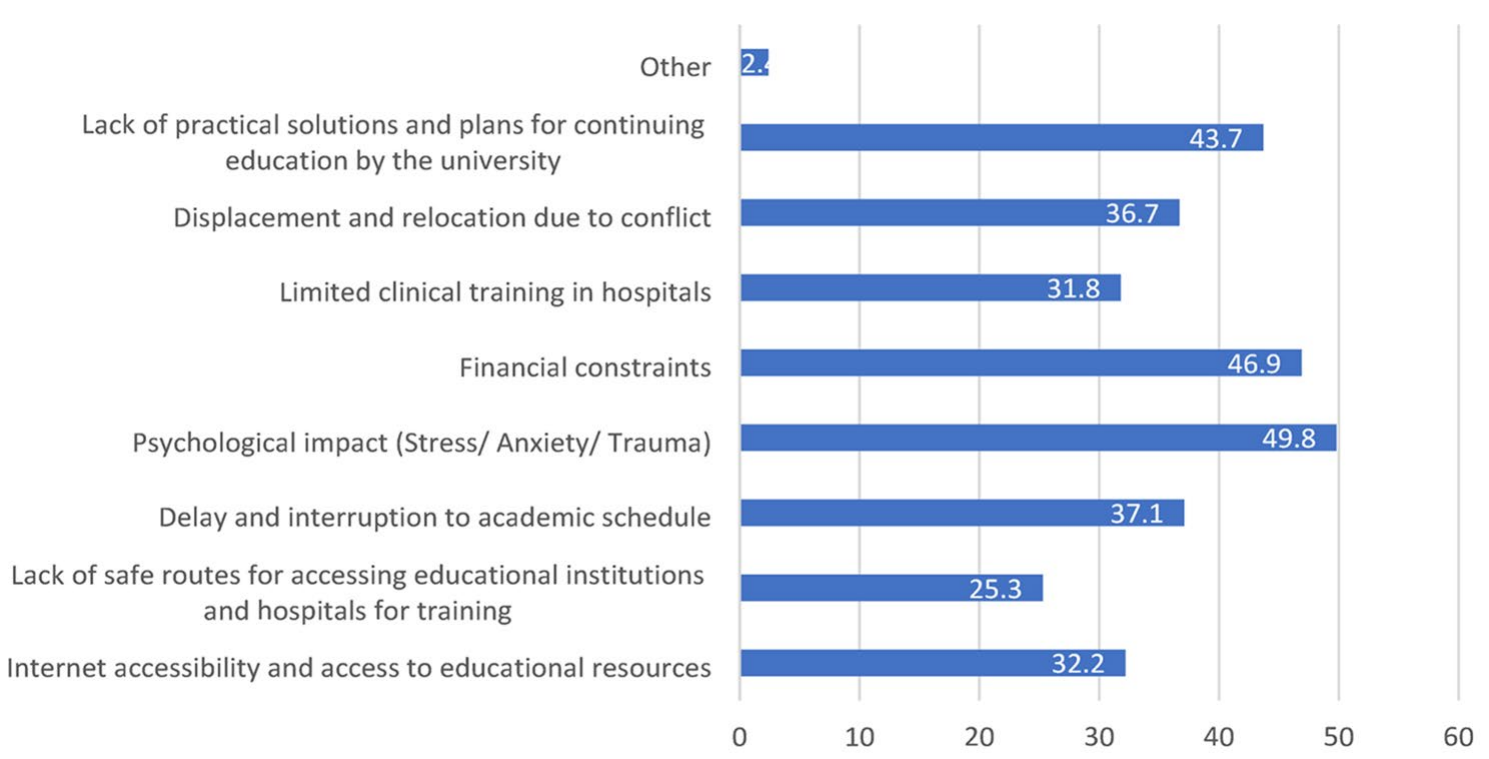
Cumulative barriers facing medical students during war period

Moreover, the participants were asked about the major barriers that currently act as an impediment to the continuation of their studies as shown in Fig. 3. The highest percentage of participants (49.8%) reported that the psychological impact imposed by the war was a major barrier. Furthermore, the financial challenges were also determined to be a major barrier to medical students (46.9%). Moreover, 43.7% of the participants stated that the lack of practical solutions by the respective universities and plans for the continuation of the education represents a major obstacle. Other reported challenges included delay and interruption to academic schedules (37.1%), displacement and relocation as a result of conflict (36.7%), and the ability to have adequate internet access (32.2%). Notably, 31.8% of participants indicated that the limited training and clinical rotations in hospitals may be a determinant factor leading to education disruption. The lack of safe routes and the limited access to educational institutions and hospitals was reported by 25.3% of participants.

**Fig. 4:**
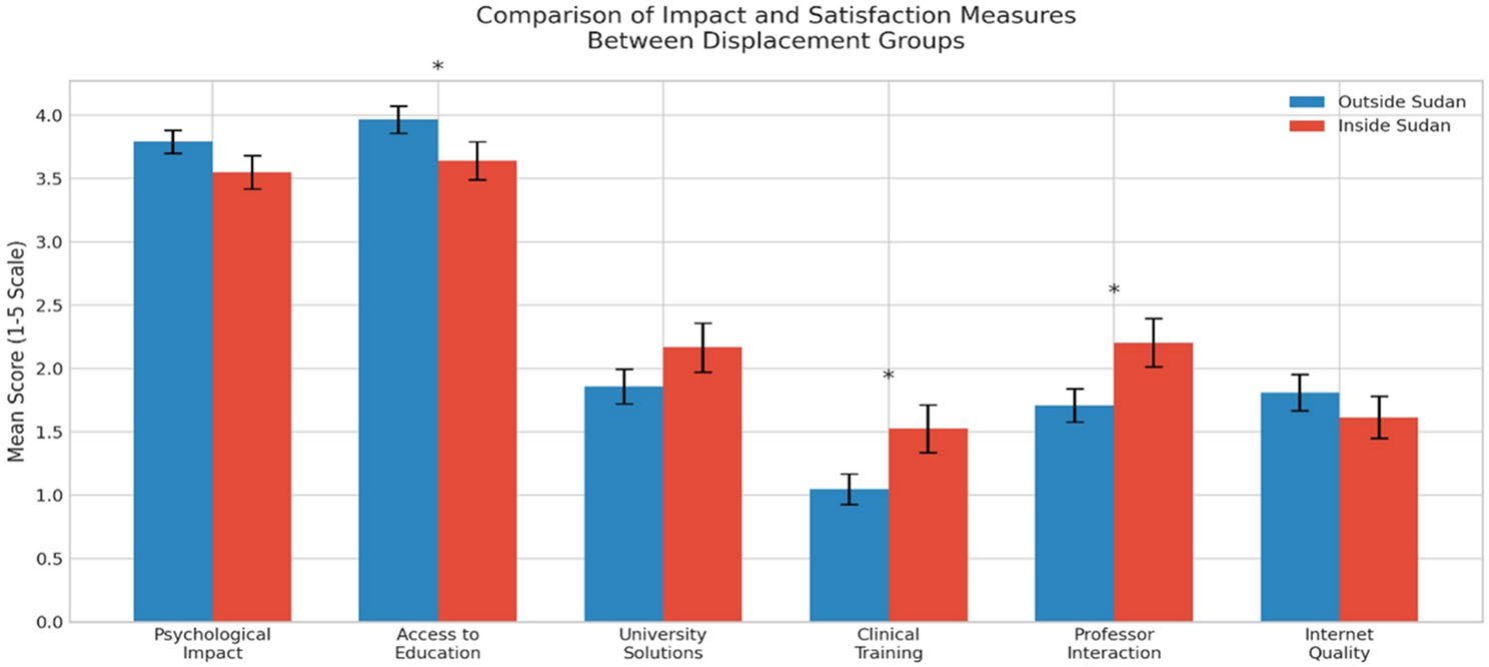
Comparison of educational and psychological outcomes by location (Inside vs. Outside Sudan)

Comparisons based on displacement status showed no significant difference in psychological impact (*p* = 0.118). Students outside Sudan reported significantly greater difficulty accessing education (*p* = 0.047) and lower satisfaction with clinical training (*p* = 0.016). Professor interaction was rated higher by students inside Sudan (*p* = 0.030). No significant differences were found in overall satisfaction with solutions or internet quality (*p* > 0.05). These findings are summarized in Table 6; Fig. 4.

**Table 6:** Comparison of educational and psychological outcomes by location (Inside vs. Outside Sudan)

Variable	Outside Sudan Mean ± SD (Median)	Inside Sudan Mean ± SD (Median)	Mean Difference	Effect Size (Cohen’s d)	U*	*p*-value
Psychological Impact	3.79 ± 1.10 (4.0)	3.55 ± 1.17 (3.0)	0.24	0.21	5068.5	0.118
Access to Education	3.97 ± 1.28 (4.5)	3.64 ± 1.32 (4.0)	0.33	0.25	4894.0	0.047*
University solutions	1.86 ± 1.67 (2.0)	2.17 ± 1.72 (2.0)	-0.31	-0.18	5167.0	0.185
Clinical Training	1.05 ± 1.48 (0.0)	1.53 ± 1.66 (1.0)	-0.48	-0.31	4726.0	0.016*
Professor Interaction	1.71 ± 1.61 (1.5)	2.21 ± 1.69 (2.0)	-0.50	-0.30	4781.5	0.030*
Internet Quality	1.81 ± 1.76 (1.0)	1.62 ± 1.48 (1.0)	0.20	0.12	5613.0	0.728

*Mann- Whitney U test

* p–value is statistically significant

Further comparisons between the participants’ demographics (age, gender, faculty and year of study), and their psychological impact, access to education, and satisfaction are presented in Table 7.

**Table 7:** Comparison of educational and psychological outcomes with demographics

Variable	Outcome	Test Statistic	*p*-value	Interpretation
Gender	Psychological Impact	Mann-Whitney U (3615.5)	0.0325*	Females reported higher psychological impact than males.
	Access to Education	Mann-Whitney U (4320.0)	0.6583	No significant gender difference in access to education.
	Satisfaction	Mann-Whitney U (2938.0)	0.0007*	Males reported higher satisfaction than females.
Academic Year	Psychological Impact	Kruskal-Wallis H (3.69)	0.5956	No significant difference in psychological impact across academic years.
	Access to Education	Kruskal-Wallis H (2.87)	0.7197	No significant difference in access across academic years.
	Satisfaction	Kruskal-Wallis H (17.6)	0.0034*	Satisfaction levels differ by academic year.
Age	Psychological Impact	Kruskal-Wallis H (0.46)	0.92	No significant difference in psychological impact across age groups.
	Access to Education	Kruskal-Wallis H (3.152)	0.368	No significant difference in access across age groups.
	Satisfaction	Kruskal-Wallis H (7.724)	0.052*	Satisfaction may differ slightly by age group.
Faculty	Psychological Impact	Kruskal-Wallis H (1.915)	0.751	No significant difference across faculties.
	Access to Education	Kruskal-Wallis H(7.606)	0.107	No significant difference across faculties.
	Satisfaction	Kruskal-Wallis H(1.312)	0.859	No significant difference across faculties.

*p–value is statistically significant

## Discussion

Regional conflict is often accompanied by proportionally disruptive outcomes in the medical sector, as evidenced across multiple global regions [13–15]. In this study from Sudan, substantial evidence demonstrated both the breakdown of long-term educational sustainability and the disruption of immediate means for education continuity. This research examined the significant consequences of conflict on 245 Sudanese medical and paramedical students enrolled in universities located in two states that were affected by war. This section explored the following key areas: displacement status and its association with the psychological and educational outcomes; the psychological impact of the conflict on students; access to medical education; overall satisfaction with the remedial actions implemented; future prospects, including dropout intentions and career shifts; and broader systemic implications for medical education during conflict.

Essentially, 93.1% of the respondents were displaced due to the ongoing conflict, 60.8% of whom migrated beyond the borders of Sudan. These findings aligned with a study conducted in Gaza by Aldabbour et al., which showed high displacement due to war, with 23.01% of the participants displaced outside Gaza, and 61.95% displaced to Gaza’s southern region [16]. Our study revealed notable disparities between students displaced within Sudan and those outside the country. Despite the geographic differences, both groups experienced high psychological distress, suggesting that the effects of the crisis were universally felt among Sudanese students regardless of location (*p* = 0.118). However, students outside Sudan reported a significantly higher impact on access to education (mean = 3.97, median = 4.5) compared to those inside Sudan (mean = 3.64, median = 4.0), with a statistically significant difference (*p* = 0.047). The significantly greater impact on students outside Sudan may indicate systemic challenges in adapting to new educational systems or the lack of institutional solutions by their universities, as reported by 43.7% of the participants in our study. This offered an insight into the concerning lack of coordination between Sudanese higher education institutions and relevant authorities to support educational continuity and standardize response strategies. As indicated in this study, the primary solutions implemented included online learning, relocation of universities to war-free states within Sudan, or the transfer to institutions abroad. However, the variability in responses among universities emphasizes the need for further research into the effectiveness and equity of these interventions.

Moreover, the overall satisfaction with university-provided solutions was low in both groups (*p* = 0.185). In terms of clinical training (*p* = 0.016) and interactions with educators (*p* = 0.030), students inside Sudan reported slightly higher satisfaction, which may reflect continued or partial physical access to institutions and local faculty. However, this difference remains inconclusive, as satisfaction levels were generally low across the board.

On the psychological front, the war had a significant impact on students, with 29.0% reporting high impact and 29.8% reporting very high impact. The war-related psychological effects remain undeniable, as evidenced by multiple studies in literature, with depression, anxiety, and post-traumatic stress being highly prevalent [16–20]. In accordance with these findings, our study reported high stress and anxiety levels (43.7%), depression (29.4%), post-traumatic stress disorder (15.5%), and distraction or loss of concentration (3.3%) among participants. Given the considerable toll on the mental health of medical students, a direct impact on academic performance may also be observed. This was demonstrated in a recent study involving Ukrainian medical students, where mental health was found to be a determining factor for both academic performance and the delivery of high-quality medical care [21]. In our study, the considerable number of psychologically distressed participants can be explained by several factors, including ongoing conflict, displacement, and uncertainty about the future exacerbated by the lack of coordinated institutional support from universities. Particularly, females reported a higher psychological impact in comparison to males, with a statistically significant difference (*p* = 0.033).

Notably, 46.1% of study participants reported a very high degree of interruption to their medical education and training. A recent review by Mahdi and Fahal highlighted the alarming impact of the conflict in Sudan, revealing that widespread destruction of educational infrastructure has led to the near-complete suspension of medical training in the country [22]. Similarly, a study by Esra et al. reported that the war significantly disrupted medical education, with 58 medical schools attacked—58.6% of which were targeted for looting or repurposed as military bases [4]. In Ukraine, disruption to educational endeavors was also evident as reported by 69% of the students [23]. These findings emphasized the devastating effects of war on medical education and the quality of medical programs. No statistically significant differences were found in access to education based on gender, age, year of study, or medical faculty (*p* ≥ 0.05).

The overall satisfaction of students with solutions implemented during the crisis was deemed low. Males reported slightly higher satisfaction levels than females (*p* = 0.0007), and satisfaction levels were also significantly associated with the academic year (*p* = 0.0034). Moreover, satisfaction level differed slightly by age group (*p* = 0.052). The findings suggested marked shortcomings in the quality of clinical and hospital training throughout the war period. The majority of respondents reported that no practical solutions were implemented regarding training. For those who received training, dissatisfaction was also predominant. The infrastructure damage and lack of safe routes may be among the top reasons for such dissatisfaction. Historical resemblance can be observed in Liberia, where the civil war severely disrupted clinical training [24]. Similarly, in Sudan, 79.3% of dental schools were subjected to military assaults, resulting in resource scarcity, particularly in dental materials, and consequently interrupting clinical training [25].

These findings indicate the need for resilient and adaptive approaches to clinical education during conflicts. Surprisingly, contrasting outcomes were observed during the war in Croatia, where medical education adapted rapidly to wartime conditions. The conflict unexpectedly fostered collaboration among medical professionals, led to the development of disaster medicine training, and resulted in increased medical research output, which enhanced both theoretical and practical components of medical education during the crisis [26]. A similar approach could be envisioned in the context of Sudan’s conflict. By integrating relevant wartime medical competencies into the curriculum, such as trauma care, management of complex injuries, triage during conflict situations, and coping strategies for psychological disorders, Sudanese medical education can be restructured to not only maintain continuity but also prepare future healthcare professionals to effectively respond to the unique challenges posed by armed conflict.

Maintaining effective communication is a mainstay for achieving optimal outcomes in healthcare [27]. Nonetheless, a strong teacher-student relationship fosters a supportive learning environment, which is crucial during times of crisis [28]. In our study, the students were primarily dissatisfied with the ability to interact with educators during the war. Likewise, dissatisfaction was prevalent regarding internet connectivity and the reliability of lecture delivery systems. These findings reflect the need for improved measures of communication and robust technological infrastructure to support education during emergencies. As per the findings of Taha et al., the integration of collaborative methodologies and innovative approaches has the potential to aid Sudanese medical institutions in navigating adversities during periods of armed conflict while simultaneously preserving the caliber of medical education [29].

A wide array of students’ prospects during the current war period was also grasped in our study. A proportion of students (19.2%) seriously considered dropping out of medical school. This growing inclination toward withdrawal was not unique to Sudan; globally, medical school dropout rates have been on the rise, particularly since the COVID-19 pandemic, due to escalating stress levels, burnout, and shifting interests toward alternative disciplines [30, 31]. In conflict-affected regions, the trend is even more pronounced. For instance, in Iraq, 26% of medical students considered dropping out amid war-related disruptions [7]. More than half of the participants also seriously contemplated starting part-time jobs, with 33.5% considering it to a very large extent, probably because of financial constraints or the need for additional support. Given the displacement and other factors imposed by war, economic struggles during wartime are undeniable. Essentially, there is a crucial need for systems of support, such as mental health centers, financial aid, and flexible learning arrangements, to help students navigate these challenges and continue their education.

Seeking scholarships or university admissions outside Sudan was also a major consideration. The need for stability, security, and better learning opportunities accounts for the major consideration of external scholarships. Moreover, nearly one-third of the students considered switching to alternative or nonmedical professions, demonstrating the impact of disrupted education and uncertainty on their future careers. Collectively, these findings signal a potential loss of medical talent and a future workforce drain, emphasizing the urgent need for strategic interventions to retain students within the healthcare education pipeline. The brain drain of medical professionals from Sudan has been surging even before the war period, since 1960 [32]. Current estimates suggest that up to 60% of Sudanese medical professionals are practicing abroad, a statistic that accentuates the chronic loss of skilled healthcare workers [33]. If unaddressed, the war may further accelerate this exodus, compounding existing health system challenges and undermining post-conflict recovery efforts. Urgent and strategic interventions are therefore necessary to support medical students, rebuild educational infrastructure, and foster retention of healthcare talent within the country.

The impact of war on medical education is profound, presenting numerous barriers that hinder the training of future healthcare professionals. Our study identified particular barriers such as lack of practical solutions (43.7%), displacement (36.7%), limited clinical training (31.8%), economic restriction (46.9%), psychological impact (49.8%), academic delays (37.1%), and availability of the internet (32.2%). Mayer et al. cited major factors such as disrupted instruction, financial issues, increased workload, and mental stress as major threats to the quality of medical education during conflicts [23]. Similarly, a scoping review by Dobiesz et al. emphasized the widespread disruptions caused by war, noting consistent challenges such as curriculum interruption, resource scarcity, and educator shortages across various conflict settings [34].

The consequences of these barriers are profound, with the effects extending to involve the broader healthcare system. The need for immediate and targeted interventions reclaiming the adaptability and continuity of medical education during the current conflict remains urgent. The paramount psychological effects on students would be mitigated by the integration of comprehensive psychological support systems, deemed critical to build resilience and improve the student’s educational experiences and outcomes.

Access to quality medical education during conflict remains severely restricted, emphasizing the importance of a collaborative, unified response. Forming partnerships among Sudanese medical schools could facilitate efficient resource sharing and foster coordinated strategies aligned with the evolving needs of the healthcare system. Furthermore, realigning medical curricula to reflect the realities of conflict will ensure that future healthcare professionals receive relevant and timely training.

Finally, and most crucially, the integration of robust, innovative teaching frameworks tailored to the current context is essential. A notable example is Karazin University in Ukraine, where the implementation of hybrid learning models during wartime significantly improved student outcomes. Sudanese institutions can draw from such experiences to guide meaningful reforms in medical education [13].

### Limitations

The study has several limitations that should be mentioned. Firstly, the study followed a convenience sampling and had a low response rate of 63.8%. This may affect the generalizability of the study findings to the medical students in Sudan. Thus, the sample may not fully represent medical students from different institutions. Moreover, internet connectivity disruptions may have resulted in a non-response bias since the study questionnaire, being distributed online, may have not reached medical students residing in areas with limited connectivity. Essentially, the war in Sudan has extended to regions beyond Khartoum and Gezira state, therefore data from other regions affected by war may have added to the significance of the findings. Notably, the lack of specific validated scales limits the precision of our psychological impact assessment. Future studies may consider incorporating validated tools, such as the PHQ-9 or GAD-7, for a more detailed analysis of psychological constructs. Moreover, overrepresentation of pharmacy students (53.9%) in the study sample may limit the generalizability of the findings to other medical disciplines, such as Medicine or Nursing, which were underrepresented. This imbalance was not addressed through weighting in the analysis, and future studies should aim for a more balanced sample to improve the external validity and applicability of the results. Finally, the study focused on insights from medical and paramedical students only. Emphasis on insights from faculty members, administrators and policymakers may have provided a comprehensive view of the challenges and institutional response during this period.

## Conclusion

In conclusion, this research elucidates the significant influence of warfare on the psychological health and academic advancement of medical students in Sudan. The results indicate elevated levels of psychological distress alongside considerable interruptions to educational and clinical training, accentuating the pressing necessity for targeted interventions. Tackling these issues necessitates a cooperative strategy that engages educational institutions, policymakers, and mental health practitioners to establish resilient frameworks that assist students amid conflict and instability. Such initiatives are essential not solely for the immediate protection of students’ well-being but also for guaranteeing the future caliber of healthcare in regions affected by war.

## Data Availability

The datasets used and/or analyzed during the current study are available from the corresponding author on reasonable request.

## Abbreviations

UMST: University of Medical Sciences and Technology
KSA: Kingdom of Saudi Arabia
UAE: United Arab Emirates
SPSS: Statistical Package for Social Sciences
PTSD: Post-Traumatic Stress Disorder
